# EMID1, a multifunctional molecule identified in a murine model for the invasion independent metastasis pathway

**DOI:** 10.1038/s41598-021-96006-2

**Published:** 2021-08-12

**Authors:** Takuya Kawata, Koji Muramatsu, Namiko Shishito, Naoki Ichikawa-Tomikawa, Takuma Oishi, Yuko Kakuda, Yasuto Akiyama, Ken Yamaguchi, Michiie Sakamoto, Takashi Sugino

**Affiliations:** 1 Division of Pathology, Shizuoka Cancer Center, Shizuoka, 411-8777 Japan; 2 Department of Pathology, Keio University School of Medicine, Tokyo, Japan; 3 Department of Cardiology, Southern TOHOKU General Hospital, Koriyama, Japan; 4 Department of Basic Pathology, Fukushima Medical University School of Medicine, Fukushima, Japan; 5 Division of Immunotherapy, Shizuoka Cancer Center Research Institute, Shizuoka, Japan; 6 Shizuoka Cancer Center Hospital and Research Institute, Shizuoka, Japan

**Keywords:** Metastasis, Molecular biology

## Abstract

EMI Domain Containing 1 (EMID1) was identified as a potential candidate metastasis-promoting gene. We sought to clarify the molecular function of EMID1 and the protein expression. Overexpression and knockdown studies using mouse tumor cell lines identified two novel functions of EMID1: intracellular signaling involving enhancement of cell growth via cell cycle promotion and suppression of cell motility, and inhibition of cell–matrix adhesion by extracellularly secreted EMID1. EMID1 deposited on the culture dish induced self-detachment of cells that overexpressed the protein and inhibited adhesion of additionally seeded cells. This multifunctional property involving both intracellular signaling and the extracellular matrix suggests that EMID1 may be a matricellular proteins. Expression analysis using immunohistochemical staining revealed expression of EMID1 that was limited to chief cells of the gastric fundic gland and β cells of the pancreatic islets in normal adult human tissues, implying cell-specific functions of this molecule. In addition, increased expression of EMID1 protein detected in some cases of human cancers implies that EMID1 might be a new therapeutic target for cancer treatment.

## Introduction

Cancer metastasis is a serious condition affecting the prognosis of patients. However, due to the diversity of metastatic processes and the molecules involved, the molecular mechanism is not fully understood. Although the canonical pathway of cancer metastasis is likely driven by invasiveness of cancer cells, we have proposed an alternative pathway called the invasion-independent pathway, in which cancer cells do not need to invade vessel walls at any step in the process of blood-borne metastasis of murine mammary tumors^1^. We established a murine model including multiple sublines with different organotropism and pathways of metastasis^2–4^. Using this model, we have identified molecules involved in this type of metastasis pathway, including secretory leukocyte protease inhibitor (SLPI)^2^ and S100A14^4^. EMI domain containing 1 (EMID1) is also a potential candidate metastasis-promoting molecule that was identified in this model system^4^.

EMID1/EMU1 is a glycoprotein identified by screening for genes that are transiently upregulated during kidney development^5^. EMID1 is a member of the Emu gene family, the members of which share an EMI domain and include EMID2, elastin microfibril interface (EMILIN) 1/2/3, and Multimelin1/2. However, few reports on EMID1 have been performed, and little is known about its molecular function, subcellular localization, and expression in adult normal tissues. In addition, there are some reports of comprehensive analysis of gene mutations and expression profiles in human cancers demonstrated that EMID1 is a potential candidate molecule associated with development or metastasis of cancers, but its molecular mechanism has not been clarified^6–8^.

The first aim of this study was to verify that EMID1 is involved in promoting metastasis in our model. We examined the effect of EMID1 overexpression or knockdown on cellular properties involved in metastasis in vitro and the metastatic ability in vivo. Our second aim was to clarify the subcellular localization of the EMID1 protein. The molecular structure of EMID1 predicts that the protein is secreted and interacts with the extracellular matrix. We analyzed the localization of EMID1 protein within the extracellular matrix of mouse tumor cells and its function in cell-matrix interaction. In addition, we aimed to clarify the distribution of EMID1 expression in adult human normal and cancer tissues. We present here the unique functions of EMID1 protein and its association with cancer metastasis.

## Results

### EMID1 expression in mouse cell lines

**Figure 1:**
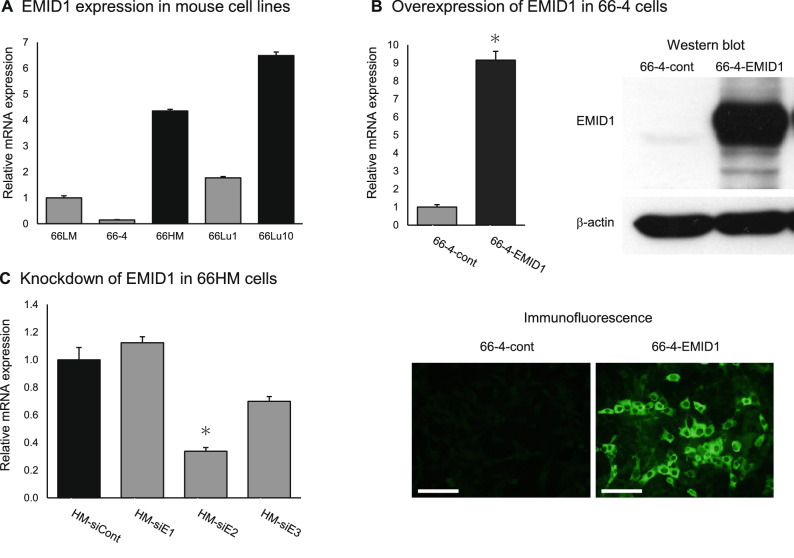
EMID1 expression in mouse cell lines. (**A**) *Emid1* mRNA expression in vitro measured with real-time qRT-PCR. Error bars, ± standard deviation (SD) (n = 3). (**B**) Effect of stable transfection of the *Emid1* expression vector on 66–4 cells. Overexpression of EMID1 was confirmed with real-time qRT-PCR (upper left), western blotting (upper right), and immunofluorescence (bottom). Error bars, ± SD (n = 3). *P < 0.05. Scale bar, 100 μm. (**C**) Knockdown effect of siRNAs for *Emid1* on 66HM cells. Relative mRNA levels of the transfectants with specific siRNAs targeting three different sequences (siE1, siE2, and siE3) were measured with real-time qRT-PCR. Error bars, ± SD (n = 3). *P < 0.05.

EMID1 is a candidate metastasis-promoting molecule identified by expression analysis using the mouse metastasis model system^1,4^. First, we reevaluated the expression level of *Emid1* mRNA in the cell lines using real-time qRT-PCR. Highly metastatic sublines, 66HM and 66Lu10, expressed *Emid1* mRNA at a higher level than low metastatic sublines (66LM, 66-4, and 66Lu1) (Fig. 1A). Next, we established gene overexpression and knockdown systems for functional analysis of EMID1 using the low metastatic subline, 66-4, and its highly metastatic counterpart, 66HM. In overexpression studies, we obtained a clone of 66-4 transfectant, 66-4-EMID1, that stably overexpresses high levels of EMID1 tagged with 6xHis. EMID1 expression in 66-4-EMID1 cells was much higher than that in the mock-transfected cells, at both the mRNA and protein levels (Fig. 1B). For knockdown studies, we first analyzed the knockdown effect of three siRNAs, siE1, siE2, and siE3, with different target sequences (Fig. 1C). siE2 showed the most effective knockdown, and we used this siRNA for the following experiments.

### EMID1 promotes cell proliferation in vitro

**Figure 2:**
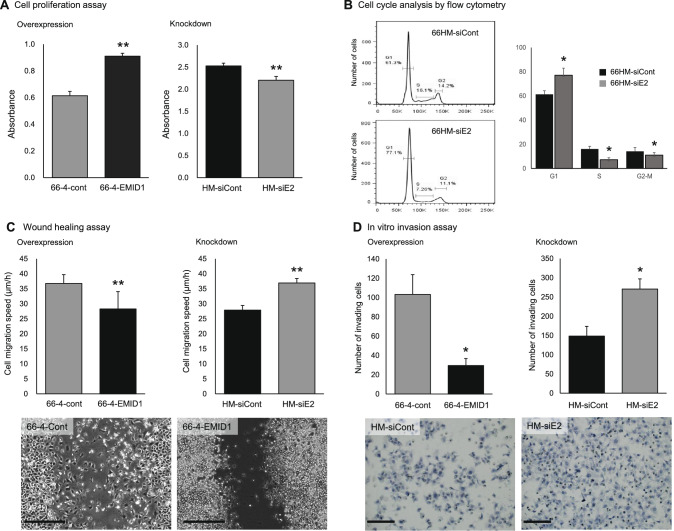
Effect of EMID1 expression on cell properties in vitro. (**A**) Cell proliferation analysis using the XTT assay. 66–4 cells stably overexpressing EMID1 and 66HM cells transfected with siRNA at 48 h were measured. Error bars, ± SD (n = 3). **P < 0.01. (**B**) Cell cycle analysis using flow cytometry. 66HM cells transfected with siE2 at 24 h were analyzed. Error bars, ± SEM (n = 3). *P < 0.05. (**C**) Cell migration analysis using the wound healing assay. The speed of wound closure for 24 h of both overexpressing and knockdown cells was evaluated (upper). Error bars, ± SD (n = 3). **P < 0.01. Phase contrast images of migrating cells (bottom). Scale bar, 500 μm. (**D**) Cell invasion analysis using the biocoat matrigel chamber. After incubation of 66–4 and 66HM transfectants for 8 and 12 h, respectively, migrating cells were counted (upper). Error bars, ± SD (n = 3). *P < 0.05. Representative image of invading cells stained with hematoxylin (bottom). Scale bar, 100 μm.

To examine the effect of EMID1 on cell proliferation, we performed three types of experiments. The first is an in vitro cell proliferation assay. The XTT assay showed that overexpression of EMID1 significantly promoted growth of 66-4 cells (Fig. 2A left), whereas knockdown of this gene significantly suppressed proliferation of 66HM cells (Fig. 2A right). These results demonstrate that EMID1 has a growth-promoting effect on murine mammary tumor cells. The second experiment is pathway analysis using comprehensive mRNA expression data analyzed using a DNA microarray. Combination analysis of differentially expressed genes in the overexpression and knockdown system showed that EMID1 may be involved in multiple pathways in cell proliferation, including the mitotic cell cycle and DNA replication (Supplementary information 2 Table S1). The third experiment is flow cytometry analysis of the cell cycle. EMID1 knockdown suppressed cell cycle progression of 66HM cells with an increase in G1 phase and reductions in S phase and G2-M phases compared to the control siRNA (Fig. 2B). These three experiments indicate that EMID1 can promote cell proliferation through cell cycle progression in mouse tumor cell lines.

### EMID1 suppresses cell motility and invasive activity in vitro

We examined the effect of EMID1 on cell motility and invasion using the wound healing assay and in vitro invasion assay, respectively. The wound healing assay revealed that overexpression of EMID1 significantly reduced the speed of wound healing by 66-4 cells compared to mock-transfected cells (Fig. 2C upper left and bottom), whereas gene knockdown significantly accelerated the speed of 66HM cells migrating into the wound (Fig. 2C upper right). These results indicate that EMID1 suppresses cell motility of murine mammary tumor cells. In the in vitro invasion assay using a matrigel-coated chamber, overexpression of EMID1 significantly reduced the number of migrating cells compared to the mock-transfected cells (Fig. 2D upper left), whereas knockdown of this gene significantly increased migrating cells (Fig. 2D upper right and bottom). These results indicate that EMID1 can suppress the invasive activity of murine mammary tumor cells.

### EMID1 overexpression does not enhance metastatic colony formation in vivo

To verify whether EMID1 promotes metastasis in vivo, we performed in vivo metastasis assay using stably overexpressing transfectants of 66-4 cells. At 8 weeks after orthotopic inoculation, overexpression of EMID1 did not increase either tumor weight or spontaneous metastasis to the lung (Supplementary information 1 Fig. S1).

### EMID1 protein deposits on the extracellular matrix

**Figure 3:**
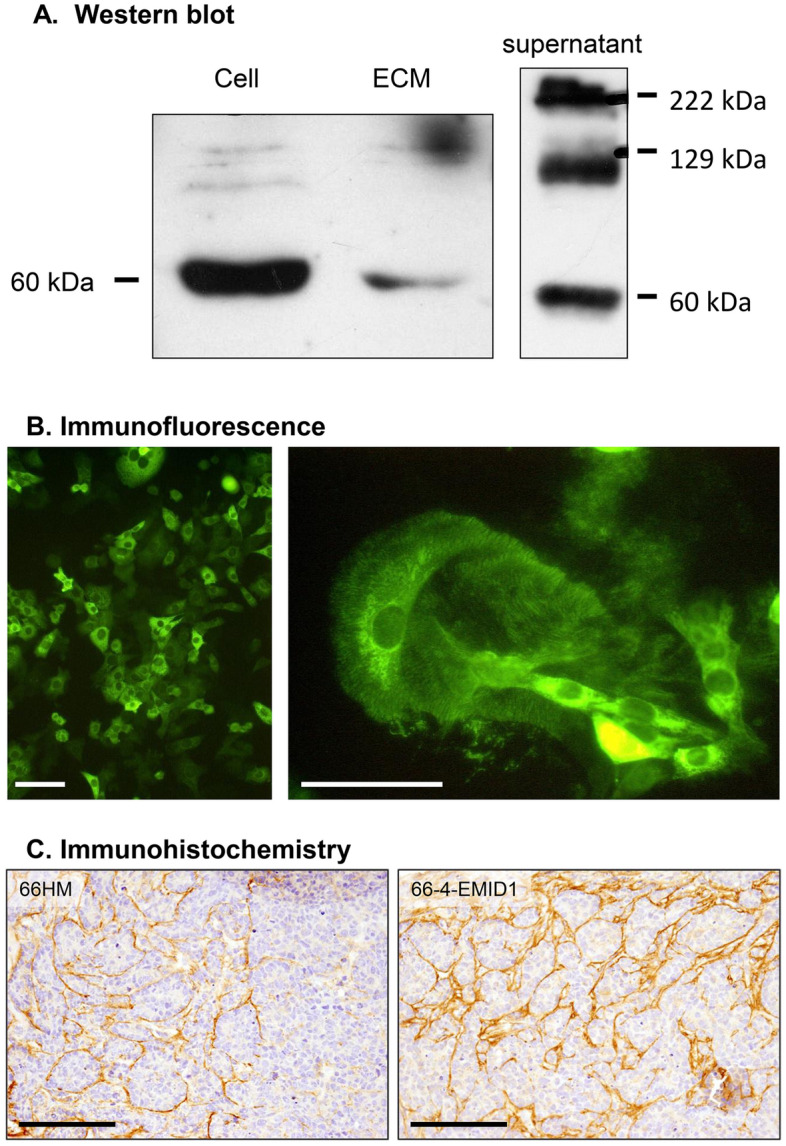
Expression and subcellular localization of EMID1 protein. (**A**) Western blots in non-reducing conditions of cell fractions of 66–4 cells transfected with the *Emid1* expression vector. (**B**) Immunofluorescent image of in vitro localization of EMID1 protein overexpressed in 66–4 cells. EMID1 protein is expressed in the cytoplasm (left) and deposited on the dish surface with brush-like structures, which are seen following cell detachment with Triton-X treatment (right). Scale bar, 50 μm. (**C**) Representative immunohistochemical image of EMID1 protein expression in vivo tumor tissue of EMID1-overexpressing 66–4 cells and 66HM cells orthotopically inoculated into the mammary fat pad of syngeneic mice. EMID1 is linearly localized around the tumor nests of both 66HM cells (left) and 66–4 cells transfected with *Emid1* expression vector (right). Scale bar, 100 μm.

The molecular structure of EMID1 including a secreted signal peptide and collagen repeats implicates its interaction with the extracellular matrix^5^. Therefore, we examined the in vitro and in vivo localization of EMID1 with a special focus on the extracellular matrix. Western blots showed that overexpressed EMID1 protein was present in the cytoplasm and dish surface as a monomer (approximately 60 kDa), and in the culture supernatant fraction as dimer and trimer (Fig. 3A). Immunofluorescence revealed that EMID1 protein was localized both in the cytoplasm and on the surface of the culture dish. After detaching the cells with Triton X-100 treatment, brush-like structures of EMID1 deposited on the dish were seen (Fig. 3B). Next, we examined the localization of EMID1 in the tumor tissue of 66-4-EMID1 cells and 66HM cells that were orthotopically inoculated into the mammary fat pad of syngeneic mice. Immunohistochemistry using mouse EMU1-179 antibody showed a linear staining pattern around the tumor nests of both cell types (Fig. 3C). These results indicate that secreted EMID1 protein can be deposited in the extracellular matrix.

### EMID1 affects the cell shape and growth morphology in vitro

**Figure 4:**
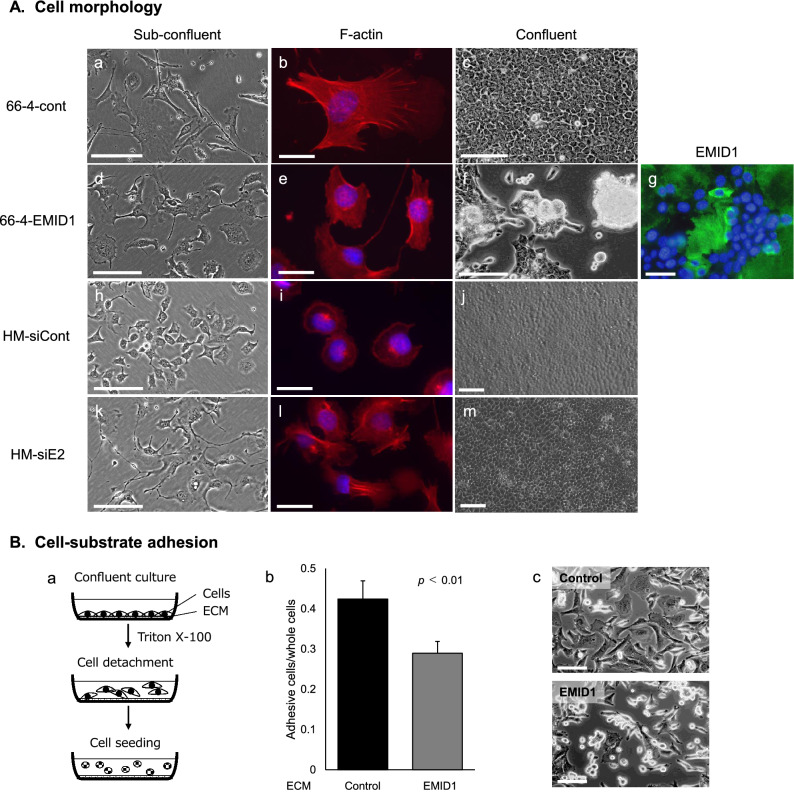
Effects of EMID1 expression on cell morphology and adhesion in vitro. (**A**) Morphological changes associated with EMID1 overexpression and EMID1 knockdown. Phase contrast images in sparse conditions (a, d, h, k). Scale bar, 100 μm. Filamentous actin formation stained with phalloidin conjugated with rhodamine (b, e, i, l). Scale bar, 50 μm. Phase contrast images (c, f, j, m) and immunofluorescence with EMID1 antibody (g) in confluent conditions. Scale bar, 100 μm. (**B**) Effect of EMID1 deposition on cell-substrate adhesion. A schematic diagram of the following experiment (a). The image was drawn using Microsoft PowerPoint 2016, https://www.microsoft.com/en-US/download/details.aspx?id=53373. The number of cells adhering to the dish in which EMID1-overexpressing 66–4 cells had been cultured was reduced compared to control cells (b). Phase contrast images of adherent cells on the dishes without and with EMID1 protein deposition (c). Scale bar, 50 μm.

Overexpression of EMID1 induced distinct morphological changes in tumor cells in vitro. In sparse conditions, 66-4-EMID1 cells exhibited more round shape with less cytoplasmic processes than 66-4-cont cells (Fig. 4A a, d). Phalloidin staining revealed that filamentous formation of F-actin in 66-4-EMID1 cells was inhibited compared with mock-transfected cells (Fig. 4A b, e). Immunofluorescence showed deposition of EMID1 protein (green) on the dish surface in the self-detached area of 66-4-EMID1 cells (Fig. 4A g). In contrast, knockdown of EMID1 induced the formation of cytoplasmic processes containing actin stress fibers in 66HM cells in sparse condition (Fig. 4A h, i, k, l), whereas in confluent condition, both knockdown cells and control cells grew as monolayer (Fig. 4A j, m). In confluent conditions, the cell sheet of 66-4-EMID1 cells detached from the culture dish and aggregated into multicellular spheroids, whereas mock transfected cells continued to adhere to the dish even after they became multilayered or piled up (Fig. 4A c, f). These results indicate that EMID1 may have two distinct effects on in vitro cell morphology in sparse and confluent conditions.

### Extracellularly deposited EMID1 suppresses cell-substrate adhesion

We tested our hypothesis that EMID1 suppresses cell-substrate adhesion. After culturing 66-4-EMID1 cells in confluent conditions, the cells were completely removed with Triton-X. Then, 66-4 parent cells were added to the culture dish, which was coated with EMID1 protein, and the number of adherent cells was counted. The number of cells that adhered to the dish was significantly reduced (Fig. 4B). This indicated that EMID1 protein deposited on the culture dish can suppress cell-substrate adhesion.

### EMID1 protein expression in normal and cancer tissues

**Figure 5:**
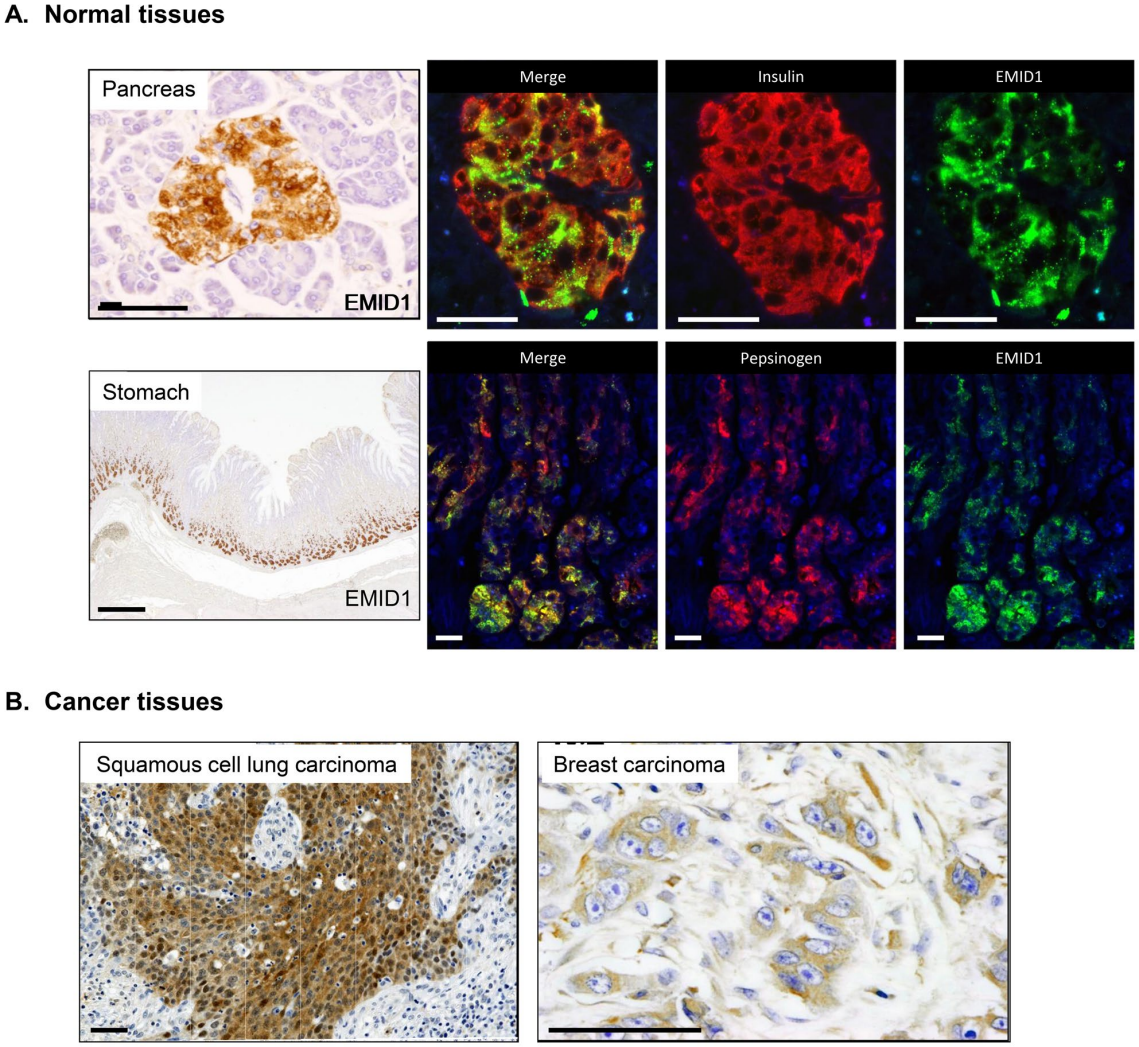
Expression of EMID1 protein in normal and cancer tissues. (**A**) EMID1 expression in human normal tissues. Immunohistochemistry and immunofluorescence show restricted expression of Emid1 protein in a pancreas islet (upper panel) and gastric fundic gland (lower panel). Scale bars indicate 50 μm and 500 μm, respectively. Emid1 (green) colocalized with insulin (red) in the pancreas and pepsinogen (red) in the stomach. Scale bar, 20 μm. (**B**) EMID1 expression in human cancer tissues. Representative immunohistochemical images of EMID1 protein expression in squamous cell lung carcinoma (left) and breast carcinoma (right). Scale bar, 50 μm.

We analyzed the tissue distribution of EMID1 protein expression in normal and cancer tissues of human body. Immunohistochemical analysis showed that EMID1 protein was exclusively and diffusely expressed in the fundic glands of the stomach and the pancreatic islets (Fig. 5A, Supplementary information 3 Table S2). Fluorescent double staining revealed colocalization of EMID1 with pepsinogen in the fundic glands and with insulin in the pancreatic islets, indicating restricted expression of EMID1 protein in the chief cells of the fundic glands and the β cells of the pancreatic islets (Fig. 5A).

Immunohistochemical analysis of human cancers revealed that various types of cancers expressed EMID1 protein in the cytoplasm (Fig. 5B, Supplementary information 3 Table S3).

## Discussion

In this study, we identified some unique features of EMID1, which has been identified as a candidate molecule for promoting cancer metastasis in the murine model. EMID1 displays multiple intracellular and extracellular functions related to metastasis in vitro, although overexpression of EMID1 did not directly promote metastasis activity in vivo (Supplementary information 1 Fig. S1). In addition, the EMID1 protein was expressed in limited types of cells in normal adult human tissues and some human cancer cases.

The molecular functions of EMID1 regarding the intrinsic properties of tumor cells are promotion of cell proliferation and suppression of invasion. Pathway analysis and cell cycle analysis in overexpression and knockdown experiments suggested that the growth-promoting effect of EMID1 is due to cell cycle progression via the intracellular growth signaling pathway (Supplementary information 2 Table S1). On the other hand, our experiments revealed that EMID1 can suppress invasive activity and cell motility. Although this effect may involve the anti-adhesion property of EMID1, incomplete cell spreading and reduced cytoplasmic processes are observed before EMID1 protein has been fully deposited on the culture dish, suggesting that these cell contact properties may be due to an intracellular effect of EMID1 on cytoskeletal formation. These results do not seem to be consistent with the general concept that the mechanism of cancer metastasis requires both cell growth and invasion. However, our results can be explained by the model of the invasion-independent metastasis pathway used in this study. In fact, we reported that SLPI, a candidate metastasis-promoting molecule identified in this model, promotes in vivo growth and spontaneous metastasis to the lung, whereas it suppresses invasive activity in vitro^2^. This is the first report showing that EMID1 affects cell growth and motility. Controversial reports have described that EMILIN1 and Multimelins, members of the Emu family to which EMID1 belongs, may be involved in cell proliferation^9–11^ and invasion^12–14^.

In addition to the intracellular functions, EMID1 has an extracellular function of inhibiting cell-matrix adhesion. Our experiments indicated that secreted EMID1 protein was deposited on the matrix beneath tumor cells and disrupted cell-matrix adhesion, which led the cells to detach from the matrix to form multicellular spheroids. Cell detachment only after confluent culture conditions suggests that a sufficient amount of EMID1 protein deposition is required for the anti-adhesion effect. Immunofluorescence showed that EMID1 deposited on the dish surface forms a unique brush-like structure in vitro, and the protein was linearly localized around tumor nests, corresponding to the basement membrane, in vivo. These findings suggest that EMID1 interacts with some components of the extracellular matrix, especially the basement membrane. Leimeister et al.^5^ reported that Emu family members can interact with different extracellular matrix components through the Emu domain to modify their functions.

This study revealed multifunctionality of EMID1 protein, with both intracellular functions: promoting cell growth and suppressing cell motility, and an extracellular function: an anti-adhesive effect on the extracellular matrix. This suggests that EMID1 may be a matricellular protein, which is a multifunctional protein that interacts with cell surface receptors or growth factors to modulate intracellular signaling and with extracellular matrix components to inhibit cell-matrix adhesion. Most matricellular proteins, such as osteopontin, tenascin-C, and periostin, are transiently expressed during development^15,16^ and in response to inflammation including wound healing, ischemia, and the foreign body response^17–19^. EMID1 is also transiently expressed in the extracellular matrix during kidney development and interacts with EMID2, which is secreted by stromal cells^5^. The only difference between EMID1 and other matricellular proteins is persistent expression in the stomach and pancreas of normal adult tissues.

In this study, we revealed exclusive expression of EMID1 protein in chief cells of gastric fundic glands and β cells of pancreatic islets in adult normal human tissues and some cases of human cancers. Interestingly, EMID1 protein in these normal and cancer tissues is localized not in the extracellular matrix but in the cytoplasm, which is unlike expression in mouse tumor cells. The reason for this is not clear, but differences in extracellular matrix configuration with which EMID1 can interact or the existence of EMID1 isoforms are assumed.

We have not yet demonstrated that EMID1 can promote cancer metastasis in this study using an overexpression method. Our previous studies identified some molecules such as SLPI and S100A14 that enhance the metastatic abilities of the low metastatic cells in our model system^2–4^. Although all of the in vitro functions of EMID1 seem to be consistent with promoting metastasis in the invasion-independent pathway, overexpression of EMID1 did not enhance spontaneous metastasis to the lung in vivo. Because EMID1 interacts with multiple molecules to modify their functions, this molecule may not act alone to promote metastasis, which is a complicated process. To clarify its association with metastasis, further studies on identification of EMID1-interacting molecules and functional analysis are needed.

In conclusion, EMID1 is differentially expressed in highly metastatic cells in a mouse mammary tumor model. Overexpression and knockdown experiments of EMID1 revealed its multifunctional characteristics in both intracellular and extracellular properties, similar to a matricellular protein. EMID1 can promote cell proliferation and suppress migration and invasion of tumor cells in vitro. In addition, EMID1 protein was secreted and deposited on the extracellular matrix to suppress cell-matrix adhesion. EMID1 protein is exclusively expressed in chief cells of the gastric fundic glands and β cells of pancreatic islets in adult human normal tissues and some human cancer cases. This is the first paper to clarify the molecular functions of EMID1 and the protein expression in human normal and cancer tissues. Further studies may clarify the physiological and pathological roles of EMID1 expression.

## Methods

### Cell lines and cell culture

The cell lines we used in this study were derived from a mouse mammary tumor, and highly metastatic (66HM and 66Lu10) and low metastatic (66LM, 66-4, and 66Lu1) sublines were selected^2–4^. These cell lines were cultured in 5% CO_2_ at 37 °C and grown in incomplete medium, which was composed of Dulbecco's Modified Eagle Medium (DMEM), low glucose (WAKO) and 10% fetal bovine serum (FBS; GIBCO).

### Plasmids and stable transfection

The full-length murine *Emid1* coding region was amplified from cDNA of 66HM cells using the forward primer 5′-ATGGGCGGCCCGCGGGCCTG-3′ and reverse primer 5′-ATGCACCACCACCATCACCATAGCTCCTCTCGCTGCGTCTCC-3′ and fused with a sequence encoding the His-tag. The construct pCIneo-*Emid1* was made by subcloning this cDNA into the pCIneo expression vector (Promega). 66-4 cells were transfected with the pCIneo-*Emid1* vector using Lipofectamine 3000 (Thermo Fisher Scientific). Stable transfectants were selected with 0.5 mg/ml G418 for 3 weeks, and strong expressers were cloned using the limiting dilution method. As a negative control, target cells were transfected with a pCIneo empty vector.

### Small interfering RNA (siRNA) transfection

Three different siRNAs against mouse *Emid1* and a negative control (Ambion) were used for transient gene knockdown in vitro. The sequences of the siRNAs are shown in Supplementary information 4 Table S4. These siRNAs were diluted in 0.1 ml Opti-MEM I medium to a final concentration of 10 nM in 24-well plates, and 1 µl lipofectamine RNAi MAX reagent (Thermo Fisher Scientific) was added to each well. After incubation for 5 min, the mixture was added to each well containing 66HM cells at 60% confluence in 0.5 ml DMEM with 10% FBS. The gene knockdown efficiency of siRNA was analyzed with quantitative real-time polymerase chain reaction (qRT-PCR).

### XTT assay

The effect of EMID1 overexpression or knockdown on in vitro cell growth was examined using an XTT assay kit (Merck). XTT (50 µl per well in a 96-well plate) labeling mixture containing 2,3-bis-(2-methoxy-4-nitro-5-sulfophenyl)-2H-tetorazolium-5-calboxanilide and phenazine methosulphate was added to 66-4 clones stably expressing EMID1 and 66HM cells at 48 h after siRNA transfection. After incubation for 24 h, absorbance at 490 nm was determined using a microtiter plate reader.

### Cell cycle analysis

The cell cycle profile was determined with flow cytometry based on the cellular DNA content using the Cell Cycle Phase Determination Kit II (Cayman Chemicals). The siRNA-transfected cells were cultured for 24 h, and 5 × 10^5^ cells were stained with 0.1% propidium iodide (Sigma Aldrich) for 30 min at room temperature. Then, they were analyzed with a flow cytometer (Canto II; BD Biosciences) according to the manufacturer's instructions.

### Wound healing assay

Cells were seeded in a 24-well plate and incubated until 90% confluent. Cell monolayers were then scratched with a p200 pipette tip. Cells migrating into the scratched region at the same points on the culture dish were visualized using microscopy at 0 and 24 h. The extent of cell migration was evaluated as the speed of wound closure at 24 h.

### In vitro invasion assay

Invasion assay was performed in a biocoat Matrigel chamber (8-µm pore; BE Biosciences) in a 24-well tissue culture plate. The upper chamber was filled with 2 × 10^5^ cells in culture medium with 10% FBS. The lower chamber was filled with 750 µl culture medium containing 10% FBS. After incubation at 37 °C for 8 and 12 hours for 66-4 and 66HM transfectants, respectively, the membranes were removed, stained with hematoxylin, and mounted on slides, and the cells on the lower side of the membrane were counted in three randomly chosen fields of view.

### Comprehensive gene expression analysis associated with EMID1 expression

Gene expression profiles associated with EMID1 were comprehensively analyzed using the microarray method. Total RNA was extracted from cultured cells using TRIZOL RNA Isolation Reagents (Thermo Fisher Scientific). Gene expression profiling was using 3D-Gene messenger RNA chip (Toray Industries) and an additional gene ontology was analyzed.

### Antibodies against mouse and human EMID1 proteins

We used three specific antibodies against mouse and human EMID1/EMU1 (IBL). The antibodies EMU1-179 and 185 were generated using 20 amino acid peptides in the middle region of mouse and human EMID1, respectively, whereas antibody EMU1-413 was raised against a 20-amino acid sequence common to the C-terminal regions of mouse and human EMID1 proteins. EMU1-185 can be used for immunohistochemistry on formalin-fixed paraffin-embedded specimens of human tissues, whereas EMU1-179 and 413 can be used for immunohistochemistry only on paraformaldehyde-fixed frozen sections. The specificity of all antibodies against EMID1 was determined by absorption tests using each EMID1 recombinant peptide; the signal of each protein was diminished in immunohistochemistry. The analysis of human tissues was approved by the Human Research Ethical Committee of Fukushima Medical University (registration number 1203). All procedures conformed to the principles outlined in the Helsinki Declaration.

### Immunofluorescence and western blot analyses

Cultured cells transfected with *Emid1-His* were plated on eight-well chamber slides for 24–48 h. Cells were fixed with 4% paraformaldehyde and permeabilized for 10 min at room temperature with 0.1% Triton-X in phosphate buffer saline (PBS). Cells were stained with Anti-His-tag mAb was conjugated with Alexa Fluor 488 (clone OGHis, 1/1000, MBL) for 30 min. Phalloidin-iFluor 594 reagent (Ab176757, 1/1000, Abcam) was used for filamentous actin (F-actin) staining. The slides were mounted in mounting medium containing 4′,6-diamidino-2-phenylindole (DAPI, Southern biotech) and observed with fluorescence microscopy (Olympus).

Western blotting was performed using cellular protein extracted with cell lysis reagent, deposited protein on the dish collected with a cell scraper, and secreted protein in serum-free medium concentrated with an iCON Concentrator 20K Pierce. Proteins (10 μg) were electrophoresed with standard sodium lauryl sulfate-polyacrylamide gel electrophoresis (SDS-PAGE) in non-reducing conditions and transferred to a polyvinylidene difluoride (PVDF) membrane (Millipore). After blocking with 5% skimmed milk for 1 h, the membrane was incubated with polyclonal rabbit antibody EMU1-179, and then incubated with anti-rabbit IgG conjugated to horseradish peroxidase (Sigma-Aldrich). The signals were visualized with enhanced chemiluminescence (ECL Advance Cytiva).

### In vivo metastasis assay

Cultured tumor cells (1 × 10^7^) suspended in 200 μl of PBS were inoculated into the mammary fat pad of 8-week-old female C3H/He mice. At 8 weeks after inoculation, animals were sacrificed by cervical dislocation, and tumor tissues and major organs were excised for counting metastatic colonies macroscopically and microscopically. All animal studies were approved by the Animal Care and Use Committee of Shizuoka Cancer Center (approval number 30-5). All methods involving animals were performed in accordance with the relevant guidelines and regulations. This study was carried out in compliance with the ARRIVE guideline.

### Cell adhesion assay

At 12 hours after seeding 1 × 10^5^ tumor cells in a 96-well plate, the cells in a confluent culture were completely removed with 0.2% Triton X-100 in PBS for 15 min at room temperature, and the well was rinsed three times with PBS. Then, 5 × 10^3^ 66-4 parent cells were added to the plate and incubated in culture medium with 10% FBS for 6 hours at 37 °C. After gently rinsing the well with PBS to remove floating cells, the remaining cells were stained with crystal violet. Absorbance at 570 nm was determined using a microtiter plate reader after elution with dimethyl sulfoxide (DMSO).

### Immunohistochemical analysis

Fresh frozen mouse sections fixed with paraformaldehyde and formalin-fixed paraffin-embedded human sections were used. Immunostaining was performed using an indirect streptavidin–biotin immunoperoxidase method (SAB-PO (M) kit; Nichirei Corp.). Formalin-fixed paraffin-embedded sections were pretreated with proteinase K (0.4 mg/ml) for 5 min for antigen retrieval. After blocking endogenous peroxidase activity with a 3% H_2_O_2_-methanol solution, the slides were incubated with primary antibodies (1/100) overnight at 4 °C, washed with PBS, and then incubated with secondary antibodies for 30 min at room temperature. Antibody localization was visualized by incubating with a secondary antibody conjugated to horseradish peroxidase for 30 min at room temperature, followed by diaminobenzidine reaction. The slides were counterstained with hematoxylin.

### Human tissue samples

An immunohistochemical study was performed using formalin-fixed, paraffin-embedded tissue specimens obtained from Shizuoka Cancer Center Hospital. Primary tumor specimens originated from the breast, lung, stomach, colon, liver, and kidney, and the corresponding normal tissues. Ten cases from each cancer type were selected. All procedures were followed in accordance with the ethical standards of the Institutional Review Board of Shizuoka Cancer Center (approval number: J2020-54-2020-1). Informed consent from enrolled patients was waived by the requirement of the approving authority.

### Statistical analysis

Unless otherwise specified, data represent the mean ± standard deviation (SD) and are representative of three independent experiments. To test for significant differences between two groups, unpaired Student’s *t* tests were used. Two-sided *p* values <0.05 were considered significant.

## Supplementary Information

Supplementary Information 1.

Supplementary Information 2.

Supplementary Information 3.

Supplementary Information 4.

## Data Availability

All data generated or analyzed during this study are included in this published article.
