# Supplementary material for: EMID1, a multifunctional molecule identified in a murine model for the invasion independent metastasis pathway
Source: Sci Rep. 2021 Aug 12;11:16372. doi: 10.1038/s41598-021-96006-2 (PMC8361151; doi:10.1038/s41598-021-96006-2)
Supplement: Supplementary file 1 — Supplementary Information 1. [file 41598_2021_96006_MOESM1_ESM.pdf]

EMID1, a multifunctional molecule identified in a murine model for the invasion independent metastasis pathway

Takuya Kawata, Koji Muramatsu, Namiko Shishito, Naoki Ichikawa-Tomikawa, Takuma Oishi, Yuko Kakuda, Yasuto Akiyama, Ken Yamaguchi, Michiie Sakamoto, Takashi Sugino*

*Correspondence to: Takashi Sugino

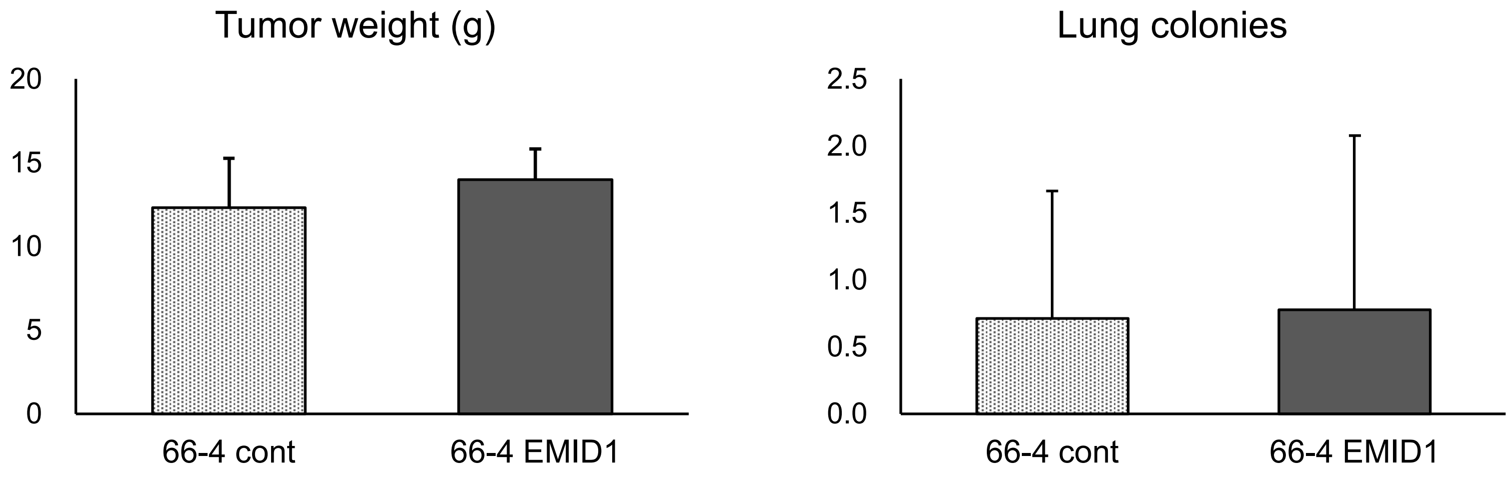

Figure S1

Tumor weight and lung metastatic colonies of EMID1-overexpressing cells inoculate in fat pad of syngenic mice. Error bars, ± standard deviation (SD)

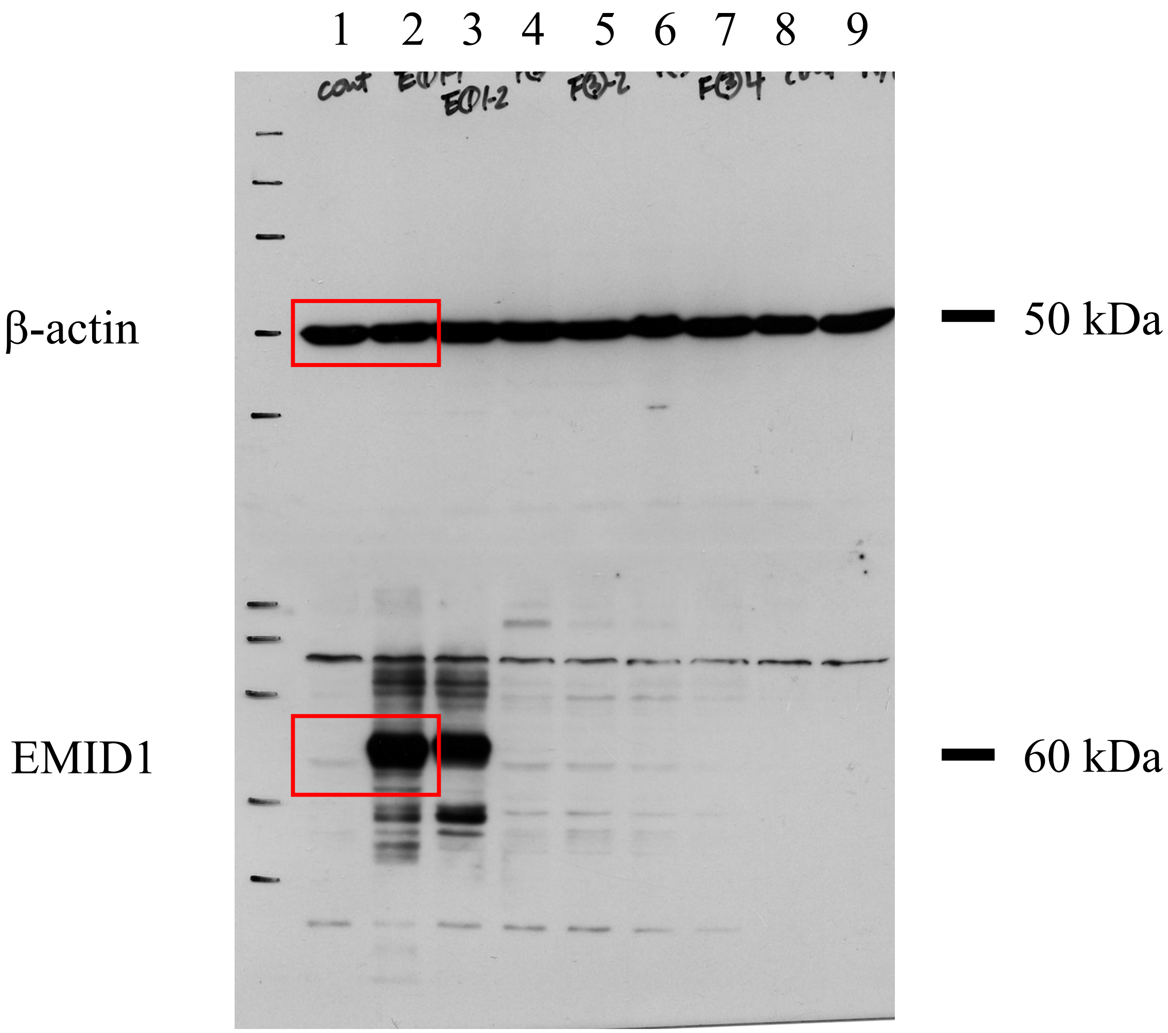

Figure S2

Extended immunoblotting image from Figure 1B.

Lane1: 66-4-cont, lane2: 66-4-EMID1 clone 1, lane3: 66-4-EMID1 clone 2

The red boxes indicate the images used in Figure 1B.

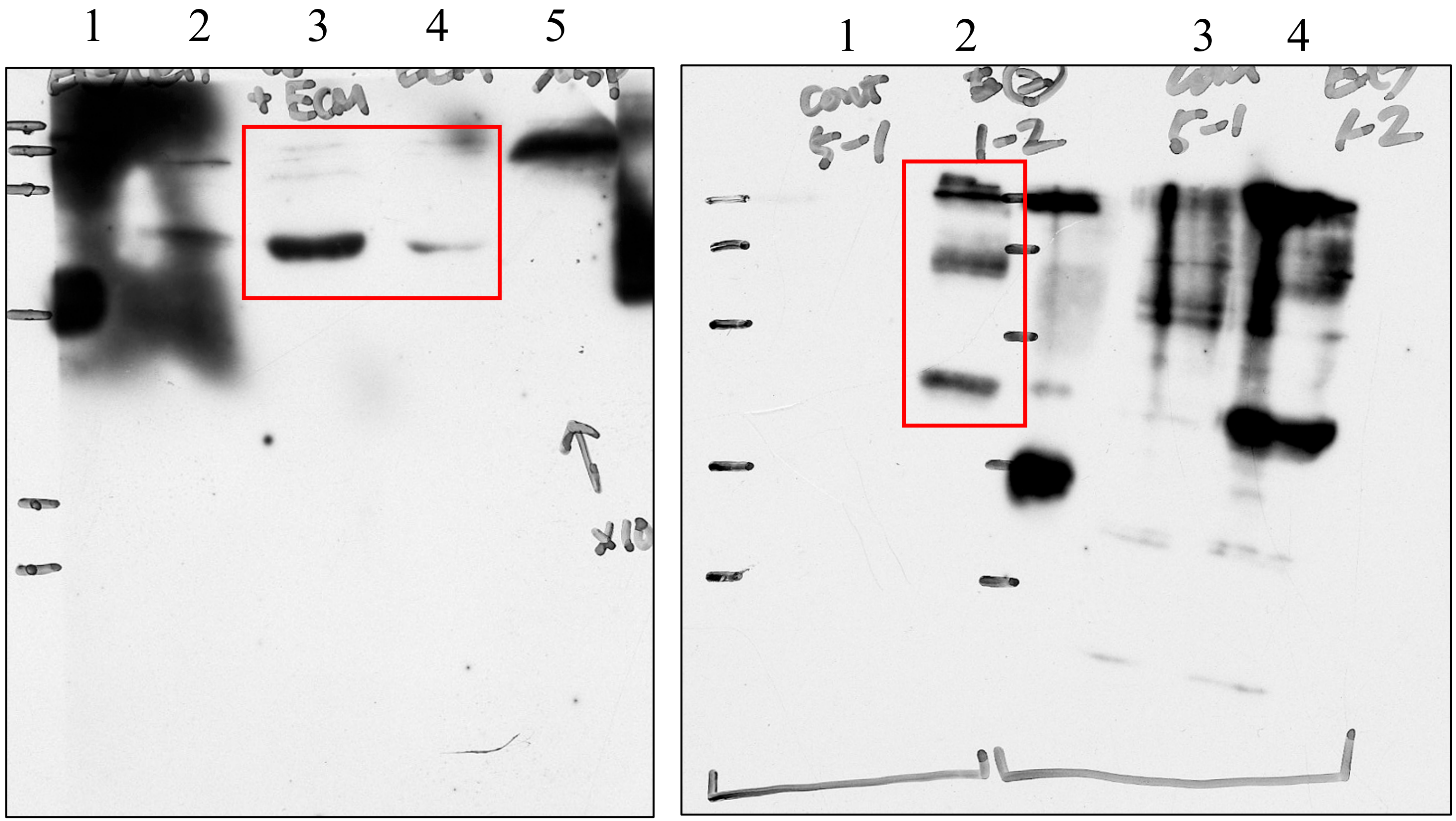

Figure S3

Extended immunoblotting images from Figure 3A.

Cells: 66-4-EMID1, antibody: anti-EMID1

Left, lane 3: cell fraction, lane 4: ECM fraction

Right, lane 2: supernatant fraction

The red boxes indicate the images used in Figure 1B.
